# Emotion Recognition Abilities in Adults with Anorexia Nervosa are Associated with Autistic Traits

**DOI:** 10.3390/jcm9041057

**Published:** 2020-04-08

**Authors:** Jess Kerr-Gaffney, Luke Mason, Emily Jones, Hannah Hayward, Jumana Ahmad, Amy Harrison, Eva Loth, Declan Murphy, Kate Tchanturia

**Affiliations:** 1 Department of Psychological Medicine, Institute of Psychiatry, Psychology and Neuroscience, King’s College London, London SE5 8AB, UK; jess.kerr-gaffney@kcl.ac.uk; 2 Centre for Brain & Cognitive Development, Birkbeck, University of London, London WC1E 7JL, UK; 3 Department of Forensic & Neurodevelopmental Sciences, Institute of Psychiatry, Psychology and Neuroscience, King’s College London, London SE5 8AB, UK; 4 School of Human Sciences, University of Greenwich, London SE10 9LS, UK; 5 Department of Psychology and Human Development, University College London, London WC1H 0AA, UK; 6 South London and Maudsley NHS Trust, National Eating Disorders Service, Psychological Medicine Clinical Academic Group, London SE5 8AZ, UK; 7 Department of Psychology, Ilia State University, Tbilisi 0162, Georgia

**Keywords:** anorexia nervosa, ASD, comorbidity, emotion recognition, attention

## Abstract

Difficulties in socio-emotional functioning are proposed to contribute to the development and maintenance of anorexia nervosa (AN). This study aimed to examine emotion recognition abilities in individuals in the acute and recovered stages of AN compared to healthy controls (HCs). A second aim was to examine whether attention to faces and comorbid psychopathology predicted emotion recognition abilities. The films expressions task was administered to 148 participants (46 AN, 51 recovered AN, 51 HC) to assess emotion recognition, during which attention to faces was recorded using eye-tracking. Comorbid psychopathology was assessed using self-report questionnaires and the Autism Diagnostic Observation Schedule–2nd edition (ADOS-2). No significant differences in emotion recognition abilities or attention to faces were found between groups. However, individuals with a lifetime history of AN who scored above the clinical cut-off on the ADOS-2 displayed poorer emotion recognition performance than those scoring below cut-off and HCs. ADOS-2 scores significantly predicted emotion recognition abilities while controlling for group membership and intelligence. Difficulties in emotion recognition appear to be associated with high autism spectrum disorder (ASD) traits, rather than a feature of AN. Whether individuals with AN and high ASD traits may require different treatment strategies or adaptations is a question for future research.

## 1. Introduction

Anorexia nervosa (AN) is a severe psychiatric disorder characterised by an intense fear of weight gain, persistent behaviour to restrict energy intake, and a disturbance in the way one’s body weight or shape are experienced [1]. Difficulties in social functioning have been identified as key factors in the development and maintenance of AN [2]. For example, before illness onset, individuals with AN report more social difficulties, fewer childhood friends, and engage in more solitary activities than healthy controls (HCs) [3,4,5,6]. During the illness, a variety of difficulties are seen, including high social anxiety, poorer social skills and social problem-solving abilities, loss of interest in social activities, and reduced social networks [7,8,9,10,11,12,13,14]. Given that interpersonal difficulties are associated with poorer outcomes in those with AN [15,16,17], it is important to understand potential underlying socio-cognitive mechanisms.

One area that has received considerable attention is emotion recognition. Given that up to two-thirds of human communication occurs through non-verbal means, recognising emotions from faces is considered key to successful social interaction [18]. Findings from studies in individuals with AN are mixed, with some reporting those with AN are significantly less accurate at inferring emotions from faces than HCs [19,20,21,22], and others reporting no differences [23,24,25,26]. A meta-analysis of 10 studies found that individuals with AN were significantly poorer at recognising basic and complex emotions relative to HCs, with small-to-medium and large effect sizes, respectively [27]. Given the effects of starvation on higher level cognitive processes, it is important to establish whether these effects may be a result of the ill state in AN. However, very few studies have examined emotion recognition performance in those recovered from AN, and results are equally mixed. While some report performance similar to that of HCs [28], others have reported poorer emotion recognition abilities, similar to those who are acutely unwell [20,29]. It is therefore not known whether potential differences in emotion recognition abilities are a result of the ill state in AN. However, one study found that emotion recognition difficulties were also present in unaffected twins of those with AN, suggesting that difficulties in this domain might represent an endophenotype for the disorder [30].

Clinical presentation of AN is associated with high levels of depression [31], anxiety [32], alexithymia [7], and autism spectrum disorder (ASD) traits [33], factors which by themselves may alter social-cognitive abilities. It is therefore possible that comorbid psychopathology might moderate emotion recognition abilities in those with AN. Although few studies have directly investigated this issue, a few have examined the impact of alexithymia. Alexithymia is a sub-clinical trait also present within the general population, describing an inability to recognise or describe one’s own emotions. When matched for levels of alexithymia, individuals with AN or bulimia nervosa (BN) have been found to show similar emotion recognition abilities to HCs [34,35], suggesting that emotion recognition difficulties may be attributable to alexithymia rather than the eating disorder (ED) per se. However, the use of mixed ED groups and small sample sizes limit interpretation of the results for AN specifically. Given the profound effects of ASD on social cognitive abilities, it is perhaps surprising that very few studies have examined the impact of ASD traits on emotion recognition in individuals with AN. Dinkler and colleagues [28] reported that individuals recovered from AN with comorbid ASD were more accurate at recognising low intensity emotional expressions than those without ASD, who did not differ from HCs. However, due to the very small sample size in the AN+ASD group (*n* = 6), analyses were treated as exploratory only. Nonetheless, these findings support the proposition that AN with or without comorbid ASD may be two qualitatively different forms of the illness [36].

Another variable that has received little attention in emotion recognition research in AN is social attention. Attending to nonverbal social cues provided by others, such as eye gaze, gestures, and facial expressions, is a necessary precursor to higher-order social cognitive abilities such as emotion recognition [37]. In typical human development, social information in the environment is highly salient, and stimuli such as faces and eyes hold particular importance [38]. This attentional bias towards social information is demonstrated from infancy, and reductions in this capacity are among one of the first signs of socio-communicative disorders such as ASD [39]. There is also emerging evidence to suggest that individuals with AN show reduced attention to faces [40] and eyes [41]. Reduced attention to facial features has been found to predict the degree of emotion recognition impairment and lower social competence in individuals with ASD [37,42,43], however only a few studies have measured attention during emotion recognition in individuals with AN. Phillipou and colleagues [44] demonstrated that while individuals with AN and HCs did not differ in their ability to recognise basic emotions, AN displayed more fixations of shorter duration to faces, indicating a “hyperscanning” strategy. Unfortunately, this study did not examine whether eye movements were associated with emotion recognition performance. In a mixed ED sample (AN or BN), Fujiwara and colleagues [35] found that difficulties in emotion recognition were predicted by less visual attention to faces in those with an ED, but not in HCs. This raises the possibility that difficulties in emotion recognition sometimes associated with EDs are a result of differences in spontaneous social attention, rather than misinterpretation of emotional displays. Finally, Dinkler et al. [28] found no differences in eye movements between those recovered from AN and HCs, and accuracy was not associated with attention to facial features. Together, these findings suggest there may be differences in the relationship between emotion recognition and attention in the acute stage of AN compared to the recovered stage or those who have never had an ED. However, studies including an acute AN group (rather than AN and BN together) are required to test this hypothesis.

The current study aimed to examine emotion recognition performance in adults in the acute and recovered stages of AN compared to HCs. It has been suggested that difficulties in this area in those with AN may be more subtle and less detectable using basic emotions [27], therefore a paradigm allowing for assessment of both basic and complex emotion recognition was selected. In order to understand why individuals with AN might display emotion recognition deficits, a secondary aim was to examine whether visual attention to faces predicted emotion recognition performance. Relatedly, a third aim was to examine whether levels of comorbid psychopathology were associated with emotion recognition performance. As well as including measures of alexithymia and ASD traits, we included depression, anxiety, and social anxiety due to their high co-occurrence with AN [8,32,45] and potential effects on social cognition [46,47,48,49,50,51]. We hypothesised that individuals with AN would be less accurate at recognising complex emotions compared to HCs, and that those recovered from AN would show intermediate levels of performance. No significant differences in basic emotion recognition were predicted. Finally, we predicted that more attention to faces, as well as lower alexithymia and ASD traits would be associated with better emotion recognition performance.

## 2. Methods

Ethical approval was obtained from the National Health Service (NHS) Research Ethics Committee (Camberwell St Giles, 17/LO/1960).

### 2.1. Participants

All participants were required to be between 18 and 55 years old and fluent in English. Exclusion criteria were a history of brain trauma or learning disability. HC participants were recruited through a King’s College London email circular and posters around campuses. Before taking part, HC participants were screened using the Structured Clinical Interview for DSM-5 Disorders, research version (SCID-5-RV) [52], to ensure they did not meet criteria for any psychiatric disorders. HCs were required to have a body mass index (BMI) between 19 and 27.

In addition to the university advertisements, participants with a lifetime history of AN were recruited through online advertisements (B-eat, call for participants, MQ Mental Health), and through two specialist NHS ED services in London. Participants were screened using the SCID-5-RV to confirm a current or past diagnosis of AN. Participants with AN were required to have a BMI ≤ 18.5 and recovered participants needed to have a BMI between 19 and 27.

### 2.2. Materials

**Figure 1:**
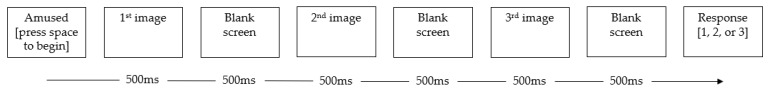
Sequence of events for an example trial of the films expression task.

**Figure 2:**
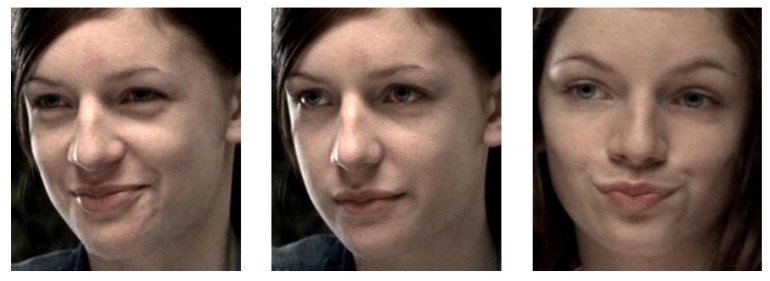
Images from an example trial [amused] of the films expression task.

The Films Expressions Task (FET) [53] is a facial emotion recognition task, modified to enable concurrent recording of eye movements. In each trial, participants are first presented with an emotion word on-screen. Three images are then presented for 500 ms each, one after another (with a 500 ms blank screen between images; see Figure 1). The height of each image was 15.7° at a viewing distance of 60 cm from the screen. The widths of each image were adjusted to ensure a correct aspect ratio and ranged from 12.4° to 21.4°. Images within each trial present the same actor displaying different emotional expressions (see Figure 2 for an example). Participants were then asked to indicate, as quickly and as accurately as they could, which of the images displayed the emotion word by pressing the corresponding key (1, 2, or 3). There were 53 trials in total (preceded by 3 practice trials). Prior to the task, participants were presented with a sheet listing the target emotion words and their definitions to ensure they were familiar with the words. A full list of the target emotion words is presented in the Supplementary Materials. Images were from films made in non-English speaking countries to reduce the probability that participants would recognise the actors.

The FET was chosen due to its depiction of naturalistic facial expressions; its inclusion of a range of both basic and complex emotions; and relatively brief presentation times. Basic and complex emotion trials were presented interleaved in a fixed random order. Foil emotional expressions were selected to be similar to the target emotion in terms of intensity of the expression and perceptual features. Development and validation of the test stimuli is presented in [53]. Dependent measures were: Accuracy (% of trials correct), mean RTs, and time spent looking at the stimuli (as a proportion of presentation time).

The Wechsler Abbreviated Scale of Intelligence, Second Edition (WASI-II) [54] measures verbal intelligence and perceptual reasoning, as well as full-scale IQ. The two subtest version was used (vocabulary and matrix reasoning).

The Autism Diagnostic Observation Schedule–2nd edition (ADOS-2), Module 4 [55] is a standardised semi-structured interview recommended for the assessment of ASD [56]. It includes a range of questions and activities designed to evoke behaviours and cognitions associated with ASD. Interviews were administered by the first author who received ADOS-2 training and met requirements for research reliability for module 4 and also attended reliability meetings throughout the study period. The revised algorithm, which was designed to more closely reflect the DSM-5 criteria for ASD was used for scoring [57]. The algorithm has two subscales: social affect and restrictive and repetitive behaviours, and total scores of 8 or more indicate possible ASD. The ADOS-2 was used in this study to identify participants with low or high ASD traits.

### 2.3. Questionnaires

The Eating Disorder Examination Questionnaire (EDE-Q) [58] measures severity of ED psychopathology. Global scores are calculated by averaging responses across items, with higher scores indicating more severe symptoms (max 6). HCs with a score of >2.7 were excluded from analyses to ensure those with possible sub-threshold ED symptoms were not included [59]. Cronbach’s alpha was 0.93.

The Hospital Anxiety and Depression Scale (HADS) [60] is a 14-item scale with two subscales: anxiety and depression. Subscale scores are interpreted as: normal (0–7), mild (8–10), moderate (11–14), and severe (15–21). Cronbach’s alpha was 0.93.

The Liebowitz Social Anxiety Scale (LSAS) [61] has two subscales: fear and avoidance of social situations. A score of 60 has been established as a cut-off indicative of social anxiety disorder (SAD) [62]. Cronbach’s alpha was 0.97.

The Social Responsiveness Scale-2nd Edition, adult self-report form (SRS-2) [63] measures symptoms associated with ASD, with higher scores (max 195) indicating more autistic symptoms. There are 5 sub-scales: social awareness, social cognition, social communication, social motivation, and restricted and repetitive interests. Cronbach’s alpha was 0.96.

The twenty-item Toronto Alexithymia Scale (TAS-20) [64] has three subscales: difficulty identifying feelings, difficulty describing feelings, and externally oriented thinking. Total scores range from 0 to 100, and cut-offs are as follows: ≤51 = no alexithymia; 52–60 = borderline alexithymia; and ≥61 = alexithymia [65]. Cronbach’s alpha was 0.90.

### 2.4. Procedure

Participants attended a testing session at the Institute of Psychiatry, Psychology & Neuroscience. After written informed consent was obtained, participants completed the FET while their eye movements were recorded using a Tobii TX300 eye-tracker. The desktop mounted eye-tracker has a sampling rate of 300 Hz, a screen resolution of 1920 × 1080, and a diagonal screen size of 23”. During tracking, infrared diodes generate reflections on the participant’s retinas and corneas. From this reflection the angular rotation of each eye is estimated. A five-point calibration procedure relates this angular rotation to corresponding x and y coordinates on the screen surface. Participants were seated approximately 60 cm from the screen. Stimulus presentation, behavioural data, and eye-tracking data were managed and recorded using custom-written MATLAB software [66].

After the FET, the first author administered the WASI-II and the ADOS-2, and the participant completed the questionnaires. Weight and height measurements were taken to calculate BMI (weight/height^2^). Participants were reimbursed £20 for their time.

### 2.5. Analysis

Histograms and Q-Q plots were inspected to check for normal distributions. Where variables were positively skewed, as was the case for RT and age data, a logarithmic transformation was applied. Homogeneity was assessed using Levene’s test. Group differences in psychopathology and demographic information were assessed using one-way ANOVAs and Tukey’s post-hoc tests, or Welch’s ANOVA with Games-Howell post-hoc tests where the assumption of homogeneity was violated.

Group differences in FET accuracy and RT were assessed with two-way mixed ANOVAs, with the within-subjects factor emotion complexity (basic or complex) and the between-subjects factor group (AN, recovered AN (REC), HC). Although analyses were conducted on log-transformed RT values, medians and interquartile range for the untransformed variable are reported for ease of interpretation, as these are similar to the geometric means. Proportion of time spent looking at faces violated the assumptions of an ANOVA (non-normal distribution, strongly negatively skewed). Therefore, group differences were assessed using the nonparametric Kruskall-Wallis test, and the effects of emotion complexity were analysed using Wilcoxon signed rank tests, with the significance level adjusted for multiple comparisons (*p* <0.01). Effects of medication on FET outcome measures were examined using independent samples *t*-tests (or a Mann-Whitney *U* test in the case of the non-normally distributed time spent looking at faces), comparing those with past or current AN who were on medication to those who were not.

Spearman’s correlations were run to examine relationships between emotion recognition performance (the primary outcome measure), demographic variables (age, IQ, BMI), psychopathology scores (EDE-Q, HADS anxiety and depression, LSAS, SRS-2, and TAS-20, ADOS-2 total), and proportion of time spent looking at faces. Variables that showed statistically significant relationships with emotion recognition performance were entered into a hierarchical regression analysis to determine which, if any, explained variance in the outcome measure.

## 3. Results

### 3.1. Demographics

In total, 148 participants were recruited (46 AN, 51 REC, 51 HC). Five HCs were subsequently excluded based on their EDE-Q scores and one REC participant was excluded due to a BMI > 27. Due to equipment failure on the day of testing, one AN and one REC participant could not complete the FET and were therefore excluded. Thus, data from 45 participants with AN, 49 REC, and 46 HC were analysed. Eye-tracking data from three HC and one REC participant was of low quality (excessive eye blinks) and was therefore excluded from analyses, however all other data (including FET accuracy and RT) from these participants was retained.

Demographic information and psychopathology scores are presented in Table 1. There were no significant group differences in age, IQ, years of education, or sex.

### 3.2. Films Expression Task

Mean emotion recognition accuracy, RTs, and proportion of time spent looking at faces across groups are displayed in Table 2. A 3 (group: AN, REC, HC) × 2 (emotion complexity: basic, complex) mixed ANOVA was computed to examine group differences in emotion recognition accuracy (% correct) for basic and complex emotions. The interaction effect was not significant, though it did reach trend level, F (2, 137) = 2.43, *p* = 0.09, ηp^2^ = 0.03. The main effect of emotion complexity was significant, F (1, 137) = 26.65, *p* <0.001, ηp^2^ = 0.16, indicating accuracy was significantly higher for basic emotions (M = 89.34%, SD = 10.92%) than complex ones (M = 85.44%, SD = 10.19). The main effect of group was not significant, F (2, 132) = 1.10, *p* = 0.34, ηp^2^ = 0.02. Accuracy (all faces) did not differ between medicated and unmedicated participants *t* (92) = 0.42, *p* = 0.67.

A 3 (group: AN, REC, HC) × 2 (emotion complexity: basic, complex) mixed ANOVA was computed to examine group differences in RTs for basic and complex emotions. The interaction effect was not significant, F (2, 132) = 0.86, *p* = 0.43, ηp^2^ = 0.01. The main effect of emotion complexity was significant, F (1, 137) = 60.72, *p* <0.001, ηp^2^ = 0.31, indicating RTs were significantly shorter for basic emotions (median = 654.14 ms, IQR = 464.68 ms) than complex ones (median = 718.29 ms, IQR = 474.55 ms). The main effect of group was not significant, F (2, 137) = 2.06, *p* = 0.13, ηp^2^ = 0.03. RTs (all trials) did not differ between medicated and unmedicated participants, *t* (92) = −1.03, *p* = 0.31.

Kruskall-Wallis tests indicated there were no significant differences between groups in the proportion of time spent looking at faces displaying basic emotions, χ^2^ (2) = 4.75, *p* = 0.09, or complex ones, χ^2^ (2) = 4.61, *p* = 0.10. Wilcoxon signed-ranks tests indicated that time spent looking at basic versus complex emotions did not significantly differ within either of the three groups (all *p* >0.01, adjusted significance level for multiple comparisons). Proportion of time spent looking at faces (overall) did not differ between medicated and unmedicated participants, *U* = 980.00, *p* = 0.57.

### 3.3. Predicting Emotion Recognition Performance

In the whole sample, emotion recognition accuracy was significantly positively associated with the proportion of time spent looking at faces (*r* = 0.17, *p* = 0.04) and IQ (*r* = 0.23, *p* = 0.01), and negatively correlated with TAS-20 (*r* = −0.18, *p* = 0.04) and ADOS-2 scores (*r* = −0.17, *p* = 0.04) (see Supplementary Materials for full table of correlations). To establish whether the relationship between accuracy and attention to faces differed across groups, correlations were run for each of the three groups separately. Proportion of time spent looking at faces significantly correlated with emotion recognition accuracy in the AN group only (*r* = 0.34, *p* = 0.02). However, a linear regression showed that proportion of time spent looking at faces did not significantly predict emotion recognition abilities in those with AN, although the association did reach trend level, F (1, 42) = 3.36, *p* = 0.07, adjusted R^2^ = 0.05. In the whole sample, a hierarchical multiple regression was run to determine if the addition of attention to faces, TAS-20, and ADOS-2 scores would improve the prediction of emotion recognition performance over group membership and IQ. The full model was significant, R^2^ = 0.22, F (6, 126) = 5.95, *p* <0.001, adjusted R^2^ = 0.18. Details of each regression model are shown in Table 3. The addition of ADOS-2 scores (model 3) led to a significant increase in R^2^ of 0.11, F (1, 127) = 17.54, *p* <0.001. The addition of proportion of time spent looking at faces (model 2) and TAS-20 scores (model 4) did not significantly add to the prediction.

### 3.4. ASD, Emotion Recognition Performance, and Attention to Faces

To further explore the relationship between ASD symptoms and emotion recognition performance, individuals with past or current AN were grouped according to whether they met the clinical cut-off for ASD on the ADOS-2 and compared with HCs. The two HCs who scored above cut-off on the ADOS-2 were excluded, due to their being too few cases to assess group differences. Thus, 44 HC, 20 lifetime AN scoring above the ADOS-2 cut-off (AN + ASD), and 74 lifetime AN scoring below the ADOS-2 cut-off (AN − ASD) were included in analyses.

**Figure 3:**
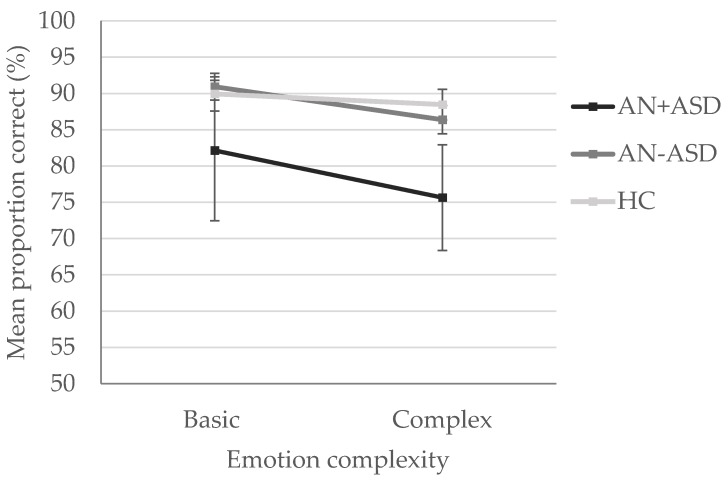
Mean proportion of correct trials on the films expression task. Error bars represent 95% confidence intervals. HC = healthy controls; AN + ASD = lifetime AN, above cut-off on the ADOS-2; AN − ASD = lifetime AN, below cut-off on the ADOS-2.

A 3 (group: AN + ASD, AN − ASD, HC) × 2 (emotion complexity: basic, complex) mixed ANOVA was computed to examine group differences in emotion recognition accuracy (Figure 3). The interaction effect was not significant, though it did reach trend level, F (2, 135) = 2.70, *p* = 0.07, ηp^2^ = 0.04. The main effect of emotion complexity was significant, F (1, 135) = 23.13, *p* <0.001, ηp^2^ = 0.15, indicating accuracy was significantly higher for basic emotions (M = 89.34%, SD = 10.99%) than complex ones (M = 85.49%, SD = 10.22). The main effect of group was also significant, F (2, 135) = 10.51, *p* <0.001, ηp^2^ = 0.14, indicating AN + ASD (M = 77.36%, SD = 16.54%) were significantly less accurate at recognising emotions than AN − ASD (M = 87.58, SD = 7.36), and HCs (M = 88.85%, SD = 6.10%), who did not differ from one another. Kruskall-Wallis tests indicated there were no significant differences across groups in the proportion of time spent looking at faces displaying basic emotions χ^2^ (2) = 2.06, *p* = 0.36, or complex ones, χ^2^ (2) = 2.92, *p* = 0.23.

## 4. Discussion

The current study aimed to examine emotion recognition abilities in those with acute AN, REC, and HCs. Contrary to our hypotheses, there were no significant differences between groups in basic or complex emotion recognition. Our prediction that emotion recognition abilities would be associated with attention to faces, as well as alexithymia and ASD traits, was partially supported. Emotion recognition accuracy was significantly positively correlated with proportion of time spent looking at faces, and negatively correlated with alexithymia (TAS-20) and autistic features (ADOS-2 scores). However, in regression analyses, only ADOS-2 scores remained a significant predictor of emotion recognition performance while controlling for IQ and group membership. A subsequent analysis demonstrated that considering acute and recovered AN together, those who scored above the clinical cut-off for ASD on the ADOS-2 were significantly less accurate at recognising emotions than those who scored below the ADOS-2 cut off and HCs. These groups did not differ in the proportion of time spent looking at faces, suggesting differences in emotion recognition abilities were not due to differences in attention. Thus, in our sample of adults with a lifetime diagnosis of AN, difficulties in emotion recognition abilities appear to be associated with high ASD traits, rather than a feature of AN.

Our findings suggest that difficulties in emotion recognition are not a feature of the socio-emotional profile hypothesised to contribute to the maintenance of AN [2]. Although our results contrast with studies showing facial emotion recognition difficulties in acute and recovered AN [19,20,29,67], several studies have failed to detect significant differences between groups [23,24,25,68,69]. It is possible that the different emotion recognition tasks used across studies contribute to the mixed results. The FET was chosen for its relative difficulty; faces are presented for 500 ms only, stimuli are naturalistic facial expressions, and a wide range of complex emotions are included in addition to the six basic emotions. Nonetheless, given that accuracy was relatively high across groups, it may be the case that there were ceiling effects in our sample. This might be due to educational levels or IQ; participants were generally highly educated and mean IQ scores across groups were higher than the population average. Indeed, in the original pilot studies of the FET, distractors were only chosen if they were misidentified as the target emotion less than 30% of the time [53], possibly resulting in the high level of accuracy seen in our sample. It should be noted that the FET has not yet been validated in a normative sample, limiting comparisons with previous literature. However, a recent study using the FET found that individuals with ASD were significantly less accurate at identifying emotions and displayed longer RTs compared to HCs [70]. Mean accuracy in the HC group (87.5% correct) was very similar to that obtained in our sample (88.64%), whereas performance in the ASD group was far lower (70.8%) than in our clinical group (acute AN = 85.2%). Although definitive conclusions cannot be made from cross-study comparisons, this pattern supports intact emotion recognition performance in acute and recovered AN.

Another explanation for the mixed results from emotion recognition studies in AN concern another of our main findings: ASD traits predicted performance rather than ED status. It could be the case that variations in ASD symptoms across study samples contribute to the mixed findings, such that group differences in mean performance may not be apparent in samples with relatively low levels of ASD traits. To further investigate this issue, future studies may benefit from looking beyond group differences in social-cognitive performance. For example, Renwick and colleagues [36] used cluster analysis to explore social- and neuro-cognitive abilities in adults with AN, including measures of set-shifting, central coherence, and theory of mind (ToM). Three clusters emerged: One characterised by average to high social- and neuro-cognitive performance; another showing mixed performance (good set-shifting, average ToM, and poor central coherence and cognitive flexibility); and a final cluster characterised by poor overall performance. The authors propose that the third cluster, which comprised 17% of participants, represented an “ASD-like” cluster. Unfortunately, no diagnostic or self-report measures of ASD were included in the study, so is in not known whether these participants met diagnostic criteria for ASD. Nonetheless, this study demonstrates that distinct sub-groups within the overall diagnosis of AN may exist, potentially with different aetiologies and developmental pathways.

Although our cross-sectional design prevents conclusions regarding the differing developmental pathways that may characterise participants in the current study, recent research presents some interesting hypotheses. For example, individuals with ASD report sensory sensitivities and a limited range of acceptable foods, often from childhood [71,72]. Further, women with ASD report high levels of eating disturbances compared to both men with ASD and neurotypical women, particularly in regards to eating rituals, sensory sensitivity to the taste, smell, and texture of food, and difficulties around eating with others [73]. These difficulties may reinforce food restriction, resulting in energy deficits and a potential trigger for the development of a clinical ED in some individuals with ASD [74]. Another possible pathway through which EDs and ASD may co-occur is via interacting influences of body dissatisfaction and gender identity. There is emerging evidence to suggest that having ASD increases one’s chances of experiencing gender dysphoria [75] and rejecting a binary gender identity [76]. In addition, qualitative reports from women with ASD often report conflict between expected feminine roles and their autistic identities [77]. At the same time, transgender individuals are at increased risk of body dissatisfaction and clinical EDs [78,79]. Specifically, restrictive eating and exercise can be a means of achieving a body congruent with one’s gender identity [80]. These differing aetiological pathways and maintenance factors for EDs are likely to have important implications for treatment.

The findings from the current study have important clinical implications. In our sample, 17.8% of acute AN and 24.5% of REC scored above the clinical cut-off on the ADOS-2, compared to 4.3% of HCs. Similar findings have been reported previously [69,81]. Interestingly, total scores on the ADOS-2 were significantly higher in acute AN than HCs, while scores in the REC group lay between the two. This pattern of results suggests that although a small proportion of ASD symptoms may be a result of starvation in acute AN, the ADOS-2 algorithm might be robust against picking up false positives. The findings from our recovered group suggest that ASD symptoms may be a stable trait, present before and after the illness in individuals with AN. Consequently, a more personalised approach to treatment in individuals with AN might be required. Treatment modules designed to improve social cognition may not be suitable for the majority of individuals with AN, however, they could prove useful in those with high ASD traits and accompanying emotion recognition difficulties. Interventions such as social skills training groups may be effective in adults and adolescents with ASD, with several studies reporting improvements in social cognition measures, social skills knowledge, and friendship quality [82,83,84,85,86]. In addition, some studies have shown improvements in mental health outcomes, suggesting a relationship between social functioning and wider mental health [87,88]. Whether such interventions might be useful for those with AN and ASD comorbidity is yet to be addressed. Thus far, interventions in AN that have incorporated emotion or social skills training, such as Cognitive Remediation and Emotion Skills Training (CREST) [89] have more heavily emphasised identifying and managing one’s own emotions rather than identifying emotions in others. Future treatment protocols may benefit from the inclusion of more extensive social cognition training specifically for those with AN and comorbid ASD.

The current study has a number of strengths. The sample size is one of the largest among eye-tracking studies in individuals with EDs (for a review, see [90]), and it is the first study to measure attention during emotion recognition in both acute and recovered AN. The inclusion of both basic and complex emotions, as well as the use of realistic photo stimuli allowed for a more ecologically valid assessment of emotion recognition abilities. However, several limitations should also be discussed. Most notably, given the short stimuli presentation times (500 ms), our paradigm only provided an assessment of early attentional engagement during emotion recognition. It may be that individuals with AN show differences in attention at later processing stages where attention is under conscious control [91]. This may explain why although attention to faces significantly correlated with emotion recognition accuracy, it did not explain a significant amount of the variance in accuracy in regression analyses. Although our quick presentation times might have replicated the fleeting facial expressions encountered in real life, future studies would benefit from measuring attention over longer periods in order to gain a better understanding of attentional processes in individuals with AN. Further, given the inclusion of both basic and complex emotion words in the FET, it is likely that verbal comprehension abilities explain significant variance in accuracy. Although we assessed associations between full-scale IQ and emotion recognition accuracy, our analyses may have benefited from including verbal IQ instead. Nonetheless, our findings show that individuals with AN have the capacity to process emotions rapidly to the same extent as HCs.

Relatedly, the short stimuli presentation times in the FET prevented a more fine-grained analysis of scan paths across the facial features. Reduced attention to the eyes has been demonstrated in acute AN during free viewing of face images, as well as during real-life social interactions [40,41]. Thus, it could be the case that our measure of overall looking times to faces was too blunt to detect group differences. Another limitation of the current study is the cross-sectional design. It cannot be ruled out that differences in socio-cognitive functioning or psychological resources contributed to the recovery of the recovered AN group. To our knowledge, no study has tested emotion recognition abilities and/or social attention over time in the same group of individuals with AN before and after recovery. Further, it must be noted that although the ADOS-2 is recommended as part of an ASD diagnostic assessment, it does not provide enough information on its own to confer a diagnosis of ASD [56]. Research using developmental measures in addition to assessing current symptoms would be informative in further defining social cognition in the AN+ASD sub-group.

To conclude, the findings of the current study suggest that emotion recognition difficulties are not a feature of the socio-emotional phenotype proposed to characterise AN. Instead, difficulties in emotion recognition appear to only be present in those with high ASD traits, independent of illness state. While it is not known whether this subgroup of individuals meets full diagnostic criteria for ASD, our findings support the notion that AN with and without high ASD traits might be two qualitatively different conditions. Whether these individuals may require different treatment strategies or adaptations to accommodate different communicative styles is a question for future research. Our results also suggest individuals in the acute and recovered stages of AN do not show any differences in attention to faces compared to HCs. However, given the limitations of our study design and the lack of research in this area, future studies should examine attention to individual facial features to expand on our findings.

## Figures and Tables

**Table 1:** Mean (SD) demographic information and psychopathology scores.

	AN (*n* = 45)	REC (*n* = 49)	HC (*n* = 46)	Test Statistics	*p*-Value	ηp^2^/*d*
Age (years) ^†^	27.04 (8.92)	26.00 (8.10)	23.87 (4.52)	F (2, 85.23) = 2.16	0.12	0.02
% female	93.5	98.0	91.1	Fisher’s exact test = 2.17	0.31	
BMI	15.75 (1.41) ^a^	21.14 (1.91) ^b^	21.69 (1.88) ^b^	F (2, 136) = 159.75	**<0.001**	0.70
Years of education	16.06 (3.07)	16.52 (2.62)	16.63 (2.45)	F (2, 136) = 0.54	0.58	0.01
IQ	110.86 (12.29)	110.16 (10.38)	113.78 (7.25)	F (2, 85.30) = 2.18	0.12	0.02
Age diagnosed ^†^	19.84 (7.39) ^a^	16.41 (3.53) ^b^	-	*t* (73.24) = 2.92	**0.01**	0.59
Illness length (years)	7.19 (7.88)	5.40 (5.65)	-	*t* (79.67) = 1.24	0.22	0.26
% on psychiatric medication	53.3 ^a^	32.7 ^b^	-	χ^2^ = 4.10	**0.04**	
EDE-Q	3.86 (1.25) ^a^	1.81 (1.52) ^b^	0.61 (0.58) ^c^	F (2, 75.81) = 125.35	**<0.001**	0.56
HADS-A	13.56 (4.51) ^a^	10.84 (5.11) ^b^	5.02 (3.09) ^c^	F (2, 87.46) = 61.90	**<0.001**	0.40
HADS-D	9.87 (4.40) ^a^	5.04 (4.02) ^b^	1.54 (1.68) ^c^	F (2, 76.50) = 77.66	**<0.001**	0.48
LSAS	68.95 (30.78) ^a^	57.08 (29.98) ^a^	27.91 (18.32) ^b^	F (2, 84.80) = 36.34	**<0.001**	0.29
SRS-2	82.43 (31.99) ^a^	70.04 (31.97) ^a^	39.23 (20.18) ^b^	F (2, 85.60) = 34.67	**<0.001**	0.28
TAS-20	58.16 (13.50) ^a^	49.81 (15.08) ^b^	37.47 (11.26) ^c^	F (2, 136) = 26.86	**<0.001**	0.29
ADOS-2						
Total	4.67 (3.94) ^a^	4.16 (4.50) ^ab^	2.70 (2.52) ^b^	F (2, 85.99) = 4.79	**0.01**	0.05
SA	4.02 (3.61) ^a^	3.71 (3.96) ^ab^	2.50 (2.38) ^b^	F (2, 86.95) = 3.48	**0.04**	0.04
RRB	0.64 (1.00) ^a^	0.45 (0.89) ^ab^	0.20 (0.58) ^b^	F (2, 86.10) = 3.82	**0.03**	0.05
% above clinical cut-off	17.8 ^a,b^	24.5 ^a^	4.3 ^b^	χ^2^ = 7.48	**0.02**	

ADOS-2: autism diagnostic observation schedule–2nd edition; AN: anorexia nervosa; BMI: body mass index; EDE-Q: eating disorder examination questionnaire; HADS-A: hospital anxiety and depression scale, anxiety subscale; HADS-D: hospital anxiety and depression scale, depression subscale; HC: healthy control; IQ: intelligence quotient; LSAS: Liebowitz social anxiety scale; REC: recovered anorexia nervosa; RRB: restrictive and repetitive behaviors; SA: social affect; SD: standard deviation; SRS-2: social responsiveness scale–2nd edition; TAS-20: twenty-item Toronto alexithymia scale. Different superscripts indicate significant differences between groups, significant *p*-values are highlighted in bold. ^†^ Variable was log transformed for analyses, original values are displayed.

**Table 2:** Mean (SD) performance and attention during the films expression task.

	AN (*n* = 45)	REC (*n* = 49)	HC (*n* = 46)
Accuracy (% correct)			
Basic emotions	88.25 (11.61)	89.80 (12.88)	89.91 (7.62)
Complex emotions	84.10 (10.79)	84.09 (11.65)	88.18 (7.14)
Reaction time (ms) ^†^			
Basic emotions	786.86 (546.32)	668.86 (415.61)	556.61 (352.43)
Complex emotions	875.69 (597.10)	703.59 (518.45)	662.13 (376.79)
Time spent looking at faces (%)			
Basic emotions	95.79 (5.85)	97.60 (2.79)	94.60 (8.23)
Complex emotions	96.54 (5.26)	97.81 (2.64)	94.37 (8.64)

AN: anorexia nervosa; HC: healthy control; REC: recovered anorexia nervosa; SD: standard deviation. ^†^ Variable was log transformed for analyses, median and IQR (of the untransformed variable) are displayed.

**Table 3:** Hierarchical regression analysis predicting emotion recognition accuracy from associated demographic variables and psychopathology scores.

	Model 1	Model 2	Model 3	Model 4
IQ	0.22 *	0.23 **	0.17 *	0.15
% of time spent looking at faces		0.17	0.10	0.11
ADOS-2			−0.35 ***	−0.31 ***
TAS-20				−0.14
R^2^	0.07	0.10	0.21	0.22

ADOS-2: autism diagnostic observation schedule–2nd edition; IQ: intelligence quotient; TAS-20: twenty-item Toronto Alexithymia Scale. Figures shown are standardized coefficients. Group membership was entered in model 1 but was not significant and not displayed here. * *p* <0.05; ** *p* <0.01; *** *p* <0.001.

## Supplementary Materials

The following are available online at https://www.mdpi.com/2077-0383/9/4/1057/s1, Table S1: Target emotions in the films expression task; Table S2: Correlations between FET accuracy, time spent looking at faces, and clinical and demographic variables in the full sample.
