# Supplementary material for: Emotion Recognition Abilities in Adults with Anorexia Nervosa are Associated with Autistic Traits
Source: J Clin Med. 2020 Apr 8;9(4):1057. doi: 10.3390/jcm9041057 (PMC7230901; doi:10.3390/jcm9041057)
Supplement: Supplementary file 1 [file jcm-09-01057-s001.pdf]

Table S1. Target emotions in the films expression task.

Basic	Happy
	Angry
	Sad
	Afraid
	Surprised
	Disgusted
Complex	Accusing
	Affectionate
	Amused
	Confident
	Defiant
	Despairing
	Disappointed
	Disbelieving
	Eager
	Furious
	Hostile
	Hurt
	Intimidating
	Joking
	Mocking
	Pleading
	Pleased
	Resentful
	Satisfied
	Shocked
	Suspicious
	Thoughtful
	Uneasy

Table S2. Correlations between FET accuracy, time spent looking at faces, and clinical and demographic variables in the full sample.												
	**FET accuracy**	**FET duration**	**Age†**	**IQ**	**BMI**	**EDE-Q**	**HADS-A**	**HADS-D**	**LSAS**	**TAS-20**	**SRS-2**	**ADOS-2**
FET accuracy	-											
FET duration	**0.17**	-										
Age†	0.03	0.03	-									
IQ	**0.23**	−0.03	0.15	-								
BMI	0.15	0.01	−0.09	0.11	-							
EDE-Q	−0.08	0.01	0.09	−0.20	**−0.51**	-						
HADS-A	−0.15	**0.21**	0.03	−0.22	**−0.47**	**0.74**	-					
HADS-D	−0.16	0.09	−0.01	−0.15	**−0.55**	**0.75**	**0.77**	-				
LSAS	−0.06	**0.21**	−0.11	**−0.24**	**−0.34**	**0.67**	**0.69**	**0.70**	-			
TAS-20	**−0.18**	0.07	**−0.19**	**−0.23**	**−0.45**	**0.63**	**0.67**	**0.70**	**0.69**	-		
SRS-2	−0.15	0.13	−0.17	**−0.29**	**−0.38**	**0.62**	**0.70**	**0.73**	**0.75**	**0.76**	-	
ADOS-2	**−0.17**	0.10	**−0.17**	−0.06	**−0.19**	**0.26**	**0.23**	**0.24**	**0.24**	**0.34**	**0.28**	-

ADOS-2: autism diagnostic observation schedule–2nd edition; BMI: body mass index; EDE-Q: eating disorder examination questionnaire; FET accuracy: proportion correct on the films expression task; FET duration: proportion of time spent looking at faces; HADS-A: hospital anxiety and depression scale, anxiety subscale; HADS-D: hospital anxiety and depression scale, depression subscale; IQ: intelligence quotient; LSAS: Liebowitz social anxiety scale; SRS-2: social responsiveness scale–2nd edition; TAS-20: twenty-item Toronto alexithymia scale. Significant correlations (p <0.05) are in bold. † Variable was log transformed for analyses.
